# Artificial intelligence for surgical care in war-torn sudan: Feasibility, barriers, and ethical perspectives from a conflict zone

**DOI:** 10.1016/j.sipas.2026.100333

**Published:** 2026-02-15

**Authors:** Alsadig Suliman, Lina SeedAhmed, Sara Hussein, Siddig Ali, Sabah Ahmed, Alkhansa Alkhider

**Affiliations:** Department of General Surgery, Sudan Medical Specialization Board, Isbitalia Street, Downtown, Khartoum, Khartoum, Sudan

**Keywords:** Artificial intelligence, Deep learning, Surgical care, Conflict zone, Low-resource settings, Sudan, Health system resilience, AI readiness, Ethical ai, Infrastructure barriers, Surgery, War zones, Sudan, Health information systems, Triage, developing countries

## Abstract

**Background:**

Artificial intelligence (AI) offers transformative potential for clinical care, yet its deployment in conflict-affected, low-resource settings remains under-researched. This study evaluates AI awareness, barriers, and readiness among Sudanese surgeons amidst the nation's ongoing armed conflict.

**Methods:**

A sequential explanatory mixed-methods design was employed. An online survey, adapted from validated instruments, assessed AI familiarity, perceived barriers, ethical concerns, and readiness among Sudanese general surgery residents and specialists. From October 2024 to June 2025, 185 completed responses were obtained from a stratified random sample of 195 eligible participants. Follow-up semi-structured interviews were conducted with 20 selected specialists. Quantitative data were analyzed using descriptive statistics, chi-square tests, and the Kruskal-Wallis test with Dunn’s post-hoc comparisons. Qualitative interviews were conducted until thematic saturation was achieved and analyzed thematically, with coding verified through inter-coder reliability.

**Results:**

While AI awareness was moderate (68.7%), practical clinical exposure (9.4%) and advanced literacy (10.3%) were notably low. Surgeons identified AI’s primary utility in training (68.2%) and perioperative decision-making (65.4%). Significant barriers included infrastructure deficits (87.6%), training gaps (79.1%), and financial constraints (72.4%). Conflict-specific challenges were acute, with 92.5% reporting severe technology shortages and 78.3% citing unreliable power/internet. Qualitative themes highlighted AI’s potential for triage and resource allocation, though concerns regarding accountability and ethical governance persisted.

**Conclusion:**

This study provides inaugural evidence on AI feasibility within an African zone. Despite infrastructure collapse, Sudanese surgeons show strong interest in AI for triage and diagnostics. To move forward, development must prioritize offline-compatible, context-adapted tools and robust governance frameworks. These findings provide a blueprint for integrating AI to bolster surgical resilience during humanitarian crises.

## Highlights

• Mixed-methods assessment of AI awareness, readiness, and perceived barriers among Sudanese surgeons working during active armed conflict.
• Findings show moderate conceptual awareness of AI but very limited hands-on exposure, with training, infrastructure, and power/connectivity constraints predominating.
• Surgeons most frequently identified potential AI applications in training, emergency triage, perioperative decision support, and resource allocation under severe constraints.
• Qualitative and quantitative integration highlights the need for offline-capable, context-adapted AI tools and clear ethical governance in fragile surgical systems.

## 1 Introduction

Artificial intelligence (AI), particularly its advanced subtypes such as deep learning, is increasingly transforming multiple fields of medicine through enhanced diagnostics, risk stratification, and clinical decision-making [1,2]. Recent studies have demonstrated the growing role of AI-based models in cancer detection, including the diagnosis and prognostication of colorectal cancer, highlighting their accuracy and clinical potential in real-world settings [3,4]. While these innovations are rapidly advancing in high-income countries, the feasibility and ethical deployment of AI-driven surgical and diagnostic tools in conflict-affected, low-resource environments remain largely unexplored [5]. Understanding how AI can function in such extreme contexts is critical to bridging the global digital divide in surgical innovation.

Sudan’s ongoing armed conflict has severely disrupted healthcare delivery, damaged hospital infrastructure, and displaced surgical teams, creating a major barrier to the adoption of advanced technologies like AI [6]. Yet, AI may offer timely support for triage, decision-making, and resource allocation—particularly in settings overwhelmed by war-related trauma. Tools such as AI-assisted trauma scoring and ICU forecasting could improve care delivery in the absence of specialized personnel [7]. While AI has shown promise in emergency surgical care in other conflict-affected settings, its feasibility within Sudan’s collapsing healthcare infrastructure has not been evaluated. This study addresses that gap by assessing awareness, perceived barriers, and readiness among Sudanese surgeons. The results will guide future AI model development, training strategies, and policy recommendations, ensuring that AI technologies are adaptable, accessible, and ethically deployed in humanitarian crisis environments [8].

To our knowledge, this is the first study in Africa—and globally during active armed conflict—to assess the feasibility of AI integration in surgical care, offering novel insights from frontline hospitals amid infrastructure collapse and workforce displacement [[9], [10], [11], [12], [13]]. This study aims to generate evidence-based insights to guide ethical, scalable AI integration in crisis-affected surgical systems—beginning with the case of Sudan.

## 2 Methods

### 2.1 Study design, sampling, and survey implementation

**Fig. 1:**
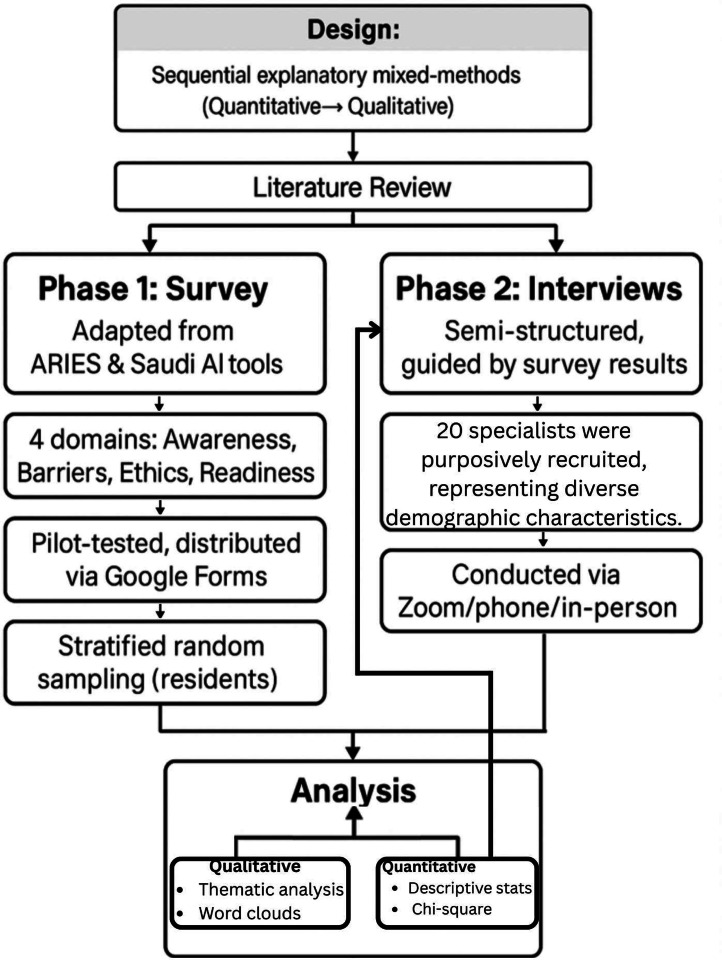
**Study Design Overview** Flowchart illustrating the sequential explanatory mixed-methods design used in the study.

We conducted a sequential explanatory mixed-methods study to assess AI feasibility in surgical care during Sudan’s ongoing conflict. The study included general surgery residents and specialists working in Sudan [14]. Quantitative survey findings informed the design of follow-up interviews to explore contextual barriers and perceptions. Sudan was selected as a case study due to its extreme resource constraints and conflict-related disruption to surgical systems. The overall study framework and mixed-methods sequence are summarized in Fig. 1.

### 2.2 Survey instrument

The questionnaire was adapted from two validated instruments: the WSES ARIES survey and a Saudi AI awareness tool [15,16]. It assessed four domains: AI familiarity, perceived barriers, ethical concerns, and readiness for adoption. Following review and pilot testing with 10 residents, the final survey was distributed online via Google Forms between October 2024 and June 2025. Detailed survey items are provided in (Supplementary material 1).

### 2.3 Sampling and recruitment

The study population included all general surgery residents registered with the Sudan Medical Specialization Board (SMSB). According to SMSB records, there were approximately 395 eligible residents across nine training patches nationwide.

**Fig. 2:**
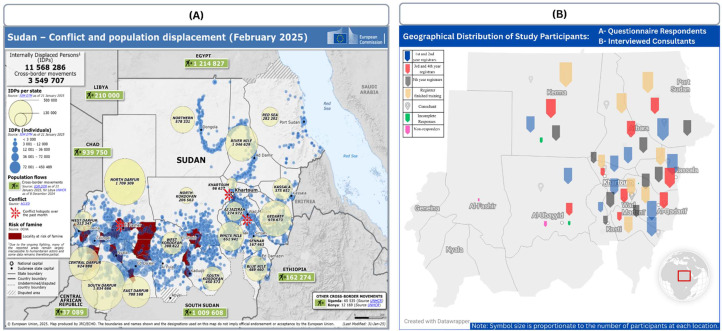
Geographical and Humanitarian Context of the Study.

The required sample size (*n* = 195) was calculated using Cochran’s formula, assuming a 95 % confidence level and a 5 % margin of error. Stratified proportional allocation was used to distribute the sample across training patches (Fig. 2). Within each stratum, participants were selected by simple random sampling from the official SMSB general surgery WhatsApp group registry, with randomization performed via Random.org. Selected participants were contacted individually through email and professional WhatsApp groups (Supplementary Material 2).

Footnote: • (A) Map of Sudan indicating conflict zones, displacement flows, and refugee numbers as of February 2025 (source: European Commission, 2025). • (B) Geographical distribution of questionnaire respondents and interviewed consultants across Sudan’s surgical training programs, showing participant representation from various conflict-affected and stable regions

The SMSB WhatsApp registries were the most current contact lists available, but they did not cover the entire eligible population. Some residents were unreachable due to displacement, relocation outside Sudan, or communication outages related to the ongoing conflict. Updated contact information for these individuals was not consistently available, so the exact number of residents outside the sampling frame is unknown.

Each participant received a unique survey link, with weekly follow-up reminders sent for three months. A formal comparison between responders and non-responders was not possible, as demographic and professional data were collected only through the survey and equivalent information for non-responders was unavailable.

### 2.4 Reachability and selection bias

Surgeons in conflict-affected areas were contacted through hospital coordinators, professional networks, and registered contacts. Non-response (≈2.6 %) occurred mainly in fully besieged or severely infrastructure-limited settings, where internet outages, unreliable electricity, and security restrictions prevented five eligible participants from receiving or completing the survey despite repeated contact attempts.

### 2.5 Handling of missing data

Survey responses were screened for completeness. Of 190 responses received, 185 (97.4 %) were fully complete, and 5 records (2.6 %) were missing more than 20 % of key variables and were therefore excluded from analysis. All remaining records were complete for the variables included in the study, so no additional handling of missing data was required.

### 2.6 Qualitative component

After analysis of the 185 completed survey responses, a total of 20 eligible specialists were approached for participation in the qualitative phase, and all 20 agreed to participate and completed the interviews (response rate 100 %).

### 2.7 Qualitative coding and reliability

Interviews were conducted by phone, Zoom, or in person using a standardized interview guide. Interviews lasted between 25 and 35 min, with a median duration of 30 min. All interviews were conducted in English; translators were not required. Audio recordings were transcribed verbatim and analyzed using NVivo (version 14). Two independent coders coded the transcripts (Supplementary material 3).

An initial codebook was developed inductively from the first five transcripts and refined through an iterative process. Inter-coder reliability was assessed on 20 % of transcripts using Cohen’s kappa (*k* = 0.78). Any coding discrepancies were resolved by consensus, with a senior investigator adjudicating when necessary

### 2.8 Bias reduction

To minimize bias, several measures were implemented: specialists were selected based on predefined inclusion criteria and demographic characteristics to reduce selection bias, with recruitment continuing until thematic saturation was achieved. The research team reviewed the participant list to ensure diversity and avoid over-representation of any subgroup. Additional steps included:

• Use of a standardized interview guide reviewed by the study steering group.
• Neutral, open-ended questioning.
• Anonymization of transcripts prior to analysis.
• Peer debriefing sessions to discuss coding and interpretation.
• Audit trail documenting all decisions during data collection and analysis.
• Member checking was performed with 6 participants (30 %) by sharing summarized findings for validation.

### 2.9 Statistical analysis

Quantitative data were analyzed using descriptive statistics and chi-square tests to assess associations between categorical or ordinal variables and AI readiness. Differences in AI understanding across residency levels were evaluated using the Kruskal–Wallis H test with tie correction. Effect size was calculated using epsilon-squared (ε²) according to the formula ε² = (*H* − *k* + 1) / (*n* − *k*). H test with tie correction; effect size was calculated using epsilon-squared, and post-hoc pairwise comparisons were conducted using Dunn’s test with Bonferroni adjustment. Potential multivariable regression models were explored to identify independent predictors of AI readiness; however, limited variability and multicollinearity among candidate predictors prevented retention of any multivariable model. Analyses were performed using SPSS version 29 (Supplementary material 4).

The qualitative phase involved semi-structured interviews conducted until thematic saturation was achieved. Transcripts were coded using NVivo version 14 with an inductively developed codebook. Inter-coder reliability was assessed on a subset of transcripts (Cohen’s kappa = 0.78). NVivo-generated visualizations supported interpretation and integration of qualitative themes.

## 3 Results

### 3.1 Baseline characteristics of participants

A total of 185 Sudanese surgeons participated in the study, working across public, private, and military/NGO hospitals (Table 1). The majority of participants were male (71.4 %), and most were aged between 30 and 34 years (38.9 %), followed by those aged 24–29 years (24.3 %).

**Table 1:** Demographic distribution of Sudanese surgical residents (*n* = 185).

Variable	Category	n	%
Age (years)	24–29	45	24.3
	30–34	72	38.9
	35–39	41	22.2
	≥40	27	14.6
Sex	Male	113	61.1
	Female	72	38.9
Years of Practice	<2 years	38	20.5
	2–5 years	61	33.0
	6–10 years	49	26.5
	>10 years	37	20.0
Workplace Type	Public hospital	149	80.5
	Private hospital	7	3.8
	Military/NGO hospital	29	15.7
Training center relocation due to war	Yes	80	43.2
	No	105	56.8

Participants represented a broad range of professional experience. Approximately one-third had 2–5 years of practice (33.0 %), while 20.5 % had less than two years and 20.0 % had more than ten years of surgical practice ensuring diverse levels of clinical exposure.

Most surgeons were employed in public hospitals (62.7 %), with smaller proportions working in military/NGO hospitals (15.7 %) and private hospitals (3.8 %). Notably, 43.2 % of participants reported that their training center had been relocated due to the ongoing conflict, reflecting substantial disruption to surgical training environments.

### 3.2 AI knowledge, perceived usefulness, and training support

Most respondents demonstrated limited advanced AI knowledge: 18.9 % could not distinguish AI terms, 46.5 % could define some, and only 24.3 % could define all, while 10.3 % reported familiarity with advanced AI concepts. Regarding AI literature, 26.5 % had not read AI articles, 20.5 % found them confusing, and 34.6 % felt comfortable with AI surgery articles; only 11.6 % could understand AI literature from computer science. Participants perceived AI as most useful for training and education (68.2 %) and perioperative decision-making (65.4 %), with lower perceived usefulness for intraoperative decision-making (47.3 %) and surgical practice (51.2 %). The top priority research areas were training/education (71.2 %), perioperative decision-making (67.1 %), and surgical vision improvement (59.3 %). A strong majority supported AI training (74.6 %) and integration into surgical education (68.2 %) (Table 2).

**Table 2:** (A) Sudanese Surgeons’ Knowledge of Artificial Intelligence Terms and Publications, the Usefulness of Artificial Intelligence in Clinical Practice for SURGERY, and Research Topics of Interest. (B) Usefulness of AI Emergency Surgery. (C) Willingness to Use and Support AI in Surgical Education.

(A)		I cannot define/ distinguish any of them		I can define/ distinguish some of them		I can define/ distinguish all of them			I am familiar with more advanced AI concepts than these
Knowledge of artificial intelligence									
Are you familiar with the following AI terms? General and narrow AI, machine and deep learning, supervised and unsupervised learning, computer vision and natural language processing		35 (18.9 %)		86 (46.5 %)		45 (24.3 %)			19 (10.3 %)
Did you read scientific articles on AI?		No	I read AI-based surgical articles, but I find them confusing	I read AI-based surgical articles, and I feel comfortable with their details		I read AI-based computer science and engineering articles and find them confusing			I read AI-based computer science and engineering articles and understand them
		49(26.5 %)	38 (20.5 %)	64 (34.6 %)		13 (7.0 %)			21(11.4 %)

(B)	Perioperative decision making	Intraoperative decision making		Improved surgical vision	Surgical practice	Training and education		Surgical robot automation	High surgery technology devices
Usefulness of AI Emergency Surgery									
I think AI applications are useful for	121 (65.4 %)	88 (47.3 %)		109 (58.9 %)	95 (51.2)	126 (68.2)		–	–
I think important AI research areas should include	124 (67.1)	82 (44.5.)		110 (59.3)	90 (48.9)	132 (71.2)		49 (26.5)	99 (53.5)

(C)									
Willingness to Use and Support AI in Surgical Education		Interested in receiving AI training					Support integrating AI into surgical education		
		138 (74.6 %)					126 (68.2 %)		

### 3.3 AI literacy, readiness, and perceived clinical utility

**Fig. 3:**
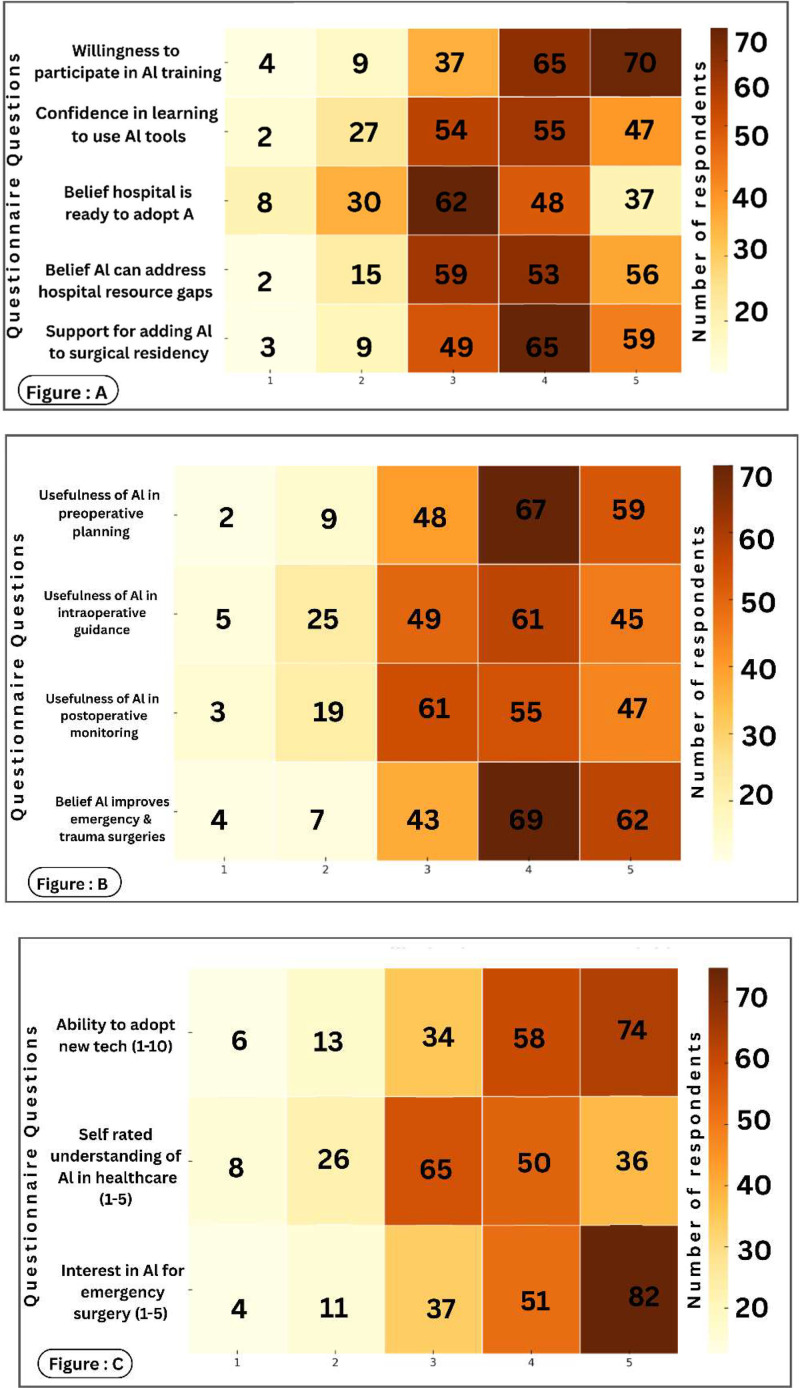
AI Perception and Readiness Heatmaps.

Awareness of AI in surgical practice was moderate (68.7 %); however, practical exposure was limited, with only 22.3 % having received formal AI training and 9.4 % reporting prior clinical use. Advanced AI literacy was low, as 10.3 % claim understood complex AI concepts and 11.6 % could interpret AI-related literature. Despite this, attitudes toward AI were largely positive: 68.2 % supported integrating AI into surgical education, 74.6 % expressed interest in AI training, and 68.1 % were willing to participate in such programs. Confidence in learning AI tools was reported by 55.1 % of respondents, while fewer (45.9 %) believed their institutions were ready for AI adoption. Perceived clinical utility was highest for emergency and trauma care (70.8 %), followed by perioperative applications, and 58.9 % agreed AI could help address hospital resource constraints (Fig. 3).

Footnote: **(**A) Readiness: Shows high willingness for AI training (70 at level 5) despite low institutional readiness (37 at level 5). (B) Utility: Highest perceived value is in emergency/trauma surgery (62 at level 5) and preoperative planning (59 at level 5). (C) Literacy: Shows a sharp interest in emergency AI (82 at level 5) despite moderate self-rated understanding (36 at level 5).

### 3.4 Barriers and conflict-related constraints to AI adoption

**Fig. 4:**
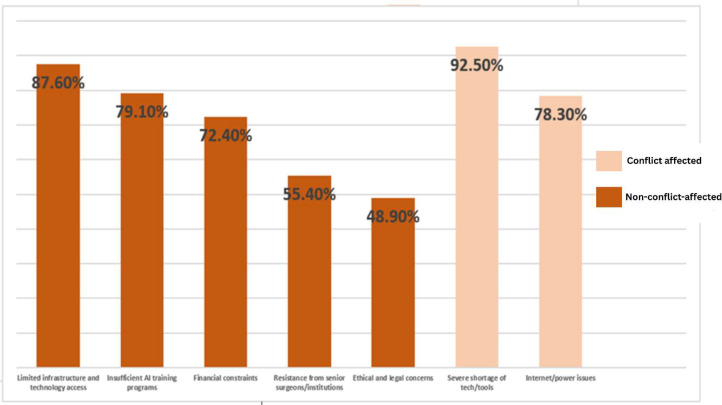
Reports of AI adoption barriers among all surveyed Sudanese surgeons (*N* = 185).

Barriers to AI adoption in Sudan included limited infrastructure, training gaps, financial constraints, ethical/legal concerns, and institutional resistance (Fig. 4), with infrastructure issues reported by 87.6 % of respondents. These challenges were amplified in conflict-affected areas or facilities receiving patients from conflict zones (43.2 %), where 92.5 % reported severe technology shortages and 78.3 % cited unreliable internet and power. Nonetheless, 66.7 % believed AI-driven triage and telemedicine could support emergency care in conflict zones, reflecting cautious optimism despite major constraints.

Footnote: • Conflict-affected: facilities located in or impacted by conflict, including those receiving war-related cases (*N* = 80). • Non-conflict-affected: facilities not reporting conflict-related impact (*N* = 105).

### 3.5 Qualitative insights: AI and surgical practice in conflict zones

**Fig. 5:**
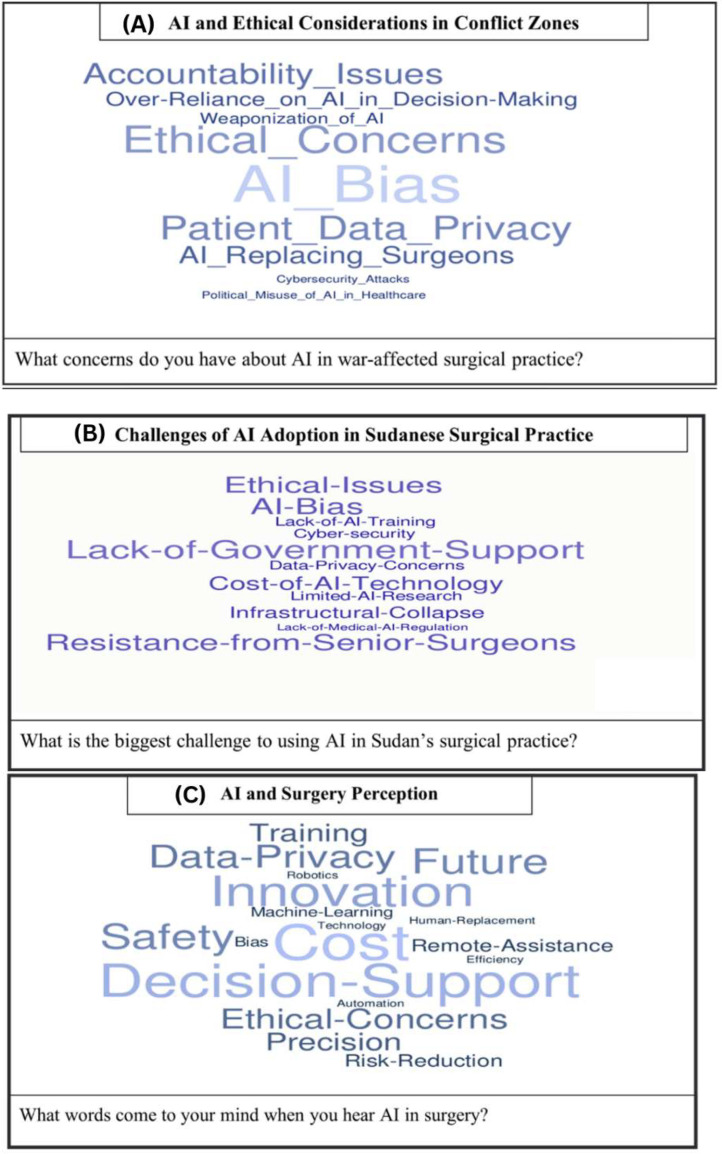
Word Clouds Depicting Key Themes from Semi-Structured Interviews with Sudanese Surgeons.

Analysis of the semi-structured interviews identified four key themes (Table 3), reflecting participants’ perspectives on the role of artificial intelligence (AI) in surgical care within conflict and resource-limited settings. Overall, there was strong optimism regarding AI’s potential to improve surgical triage and resource allocation, alongside notable concerns related to training deficiencies and ethical accountability (Fig. 5).

**Table 3:** Key Themes from Semi-Structured Interviews on AI in Surgical Care and Corresponding Codebook Mapping.

Theme	Codebook Code(s)	Description	Frequency (n, %)	Key Insight	Representative Quotation
AI as a triage and resource allocation tool	B2: Priority Areas for AI; B1: Perceived Benefits	AI could enhance crisis decision-making and optimize scarce resources	20 (100 %)	All participants believed AI could improve triage and resource allocation in high-pressure scenarios	“In mass casualty situations, AI could help us prioritize patients faster and allocate scarce resources more rationally.” (General Surgeon, Public Hospital, P01)
Lack of training	C2: Training and Human Capacity	Insufficient AI education and hands-on training	18 (90 %)	Most participants cited absence of formal training as a critical barrier	“We are expected to use advanced technology, but no one has trained us on how AI systems actually work.” (General Surgeon, Public Hospital, P12)
Ethical concerns about AI reliability and liability	D1: Ethical Concerns; D3: Accountability and Liability	Worries about over-reliance, accountability, and legal responsibility	15 (75 %)	Many participants expressed concern about AI recommendations in life-threatening situations	“If an AI system makes a wrong recommendation and a patient dies, who is responsible—the surgeon or the software?” (General Surgeon, Public Hospital, P15)
Need for AI-assisted trauma diagnostics	B2: Priority Areas for AI; A2: Current Use of AI/Technology	AI to supplement unavailable radiology/pathology services	20 (100 %)	All participants highlighted AI as critical in resource-limited or rural conflict settings	“AI-based imaging or decision support could be life-saving where CT scans or pathologists are simply not available.” (Surgeon, Rural Hospital, P19)

Footnote: Themes were derived from coding semi-structured interviews using the definitions provided in Supplementary File 4 (Codebook). Frequencies indicate the number and proportion of participants whose responses were categorized under each theme. Not all codes were represented as separate themes in the final analysis; some were integrated into broader themes based on conceptual similarity and frequency of mention.

Footnote: Word clouds illustrate the most frequently cited terms from interviews regarding artificial intelligence (AI) in surgical care within conflict-affected settings. Prominent words highlight participants’ primary concerns, perceived benefits, and barriers to AI adoption. Panels depict: (A) AI and ethical considerations in conflict zones; (B) Challenges to AI adoption in Sudanese surgical practice; (C) Perceptions of AI in surgical care.

All participants highlighted AI as a valuable tool for triage and crisis decision-making. One trauma surgeon working in a conflict-affected public hospital stated, *“In mass casualty situations, AI could help us prioritize patients faster and allocate scarce resources more rationally”* (Surgeon, Public Sector, P01). Similarly, another participant noted that AI-driven decision support could reduce cognitive burden during emergencies, *“especially when human judgment is overwhelmed”* (Surgeon, military Hospital, P07).

A dominant barrier to implementation was the lack of formal training in AI applications. As one senior surgeon explained, *“We are expected to use advanced technology, but no one has trained us on how AI systems actually work or how reliable they are”* (Consultant Surgeon, public Hospital, P12). This concern was echoed by others who emphasized that insufficient training limits trust and adoption in clinical practice.

Ethical concerns regarding reliability, accountability, and medico-legal responsibility were also frequently raised. Participants expressed apprehension about over-reliance on AI in high-stakes environments. One respondent remarked, *“If an AI system makes a wrong recommendation and a patient dies, who is responsible—the surgeon or the software?”* (Surgeon, Public Hospital, P15). Such concerns underscored the need for clear governance frameworks and human oversight.

Finally, several participants emphasized the urgent need for AI-assisted diagnostic tools in trauma care, particularly in facilities lacking radiology or pathology services. A surgeon working in a rural conflict zone stated, *“AI-based imaging or decision support could be life-saving where CT scans or pathologists are simply not available”* (Surgeon, Conflict Zone Hospital, P19). This theme highlights AI’s perceived role in mitigating structural deficiencies in underserved settings. Participant identifiers used in quotations (P0*“*N*”*) correspond to the anonymized participant list and coding framework provided in Supplementary Materials 5.

### 3.6 AI understanding across surgical training levels

A Kruskal-Wallis H test was conducted to examine differences in self-reported AI understanding across three residency levels. Medians and interquartile ranges were: 1st–2nd year 2 (IQR 1–3), 3rd–4th year 5 (IQR 4–6), and 5th year 8 (IQR 7–9) (Table 4). Tie correction was applied. The test indicated significant differences between groups, H(2) = 156.00, *p* < 0.001, with a large effect size (ε² = 0.85), reflecting a substantial separation in AI understanding across residency levels.

**Table 4:** Differences in AI Understanding Across Residency Levels.

A. AI Understanding Across Residency Levels			
Residency Level		Median AI Understanding	IQR (25th–75th percentile)
1st–2nd year (*n* = 56)		2	1–3
3rd–4th year (*n* = 63)		5	4–6
5th year (*n* = 66)		8	7–9

B. Dunn Post-Hoc Pairwise Comparisons			
Comparison	Bonferroni-adjusted p		
1st–2nd vs 3rd–4th	< 0.001		
1st–2nd vs 5th	< 0.001		
3rd–4th vs 5th	< 0.001		

Fote Note: Kruskal-Wallis test H(2) = 156.00, *p* < 0.001, tie correction applied. Effect size ε² = 0.85, calculated as (*H* − *k* + 1)/(*n* − *k*). Post-hoc pairwise comparisons were conducted using Dunn’s test with Bonferroni adjustment.

Post-hoc pairwise comparisons using Dunn’s test with Bonferroni adjustment confirmed that 5th-year residents reported significantly higher AI understanding than both 1st–2nd year (*p* < 0.001) and 3rd–4th year residents (*p* < 0.001), while 3rd–4th year residents scored significantly higher than 1st–2nd years (*p* < 0.001). These findings highlight a pronounced training-level-dependent gap in AI literacy, emphasizing the need for early integration of AI education into the Sudan Medical Specialization Board curriculum.

### 3.7 Integration of quantitative and qualitative findings

To integrate the quantitative and qualitative findings, we mapped the most frequent survey barriers and readiness indicators onto the themes identified from interviews. The qualitative data contextualized the quantitative results, illustrating how infrastructure collapse, training gaps, financial constraints, and conflict-related power and internet outages directly limit AI adoption in surgical practice. These integrated findings are summarized in (Table 5).

**Table 5:** Integration of Quantitative and Qualitative Findings.

Key Quantitative Finding ( %)	Qualitative Theme / Explanation	Representative Quote
87.6 % reported limited infrastructure as a barrier	Infrastructure collapse prevents AI implementation	“We cannot rely on AI because power goes off 4–5 times per day.” (Surgeon, Public Hospital, P02)
79.1 % reported lack of training	No AI education in residency; surgeons lack trust in AI	“No one taught us how AI works, so we don’t trust it.” (Surgeon, Public Hospital, P12)
72.4 % reported financial constraints	Hospitals cannot afford AI devices or software licenses	“Even if AI tools exist, we cannot afford them or the internet.” (Surgeon, NGO Hospital, P05)
92.5 % in conflict zones reported unreliable power	AI needs offline solutions and low bandwidth	“AI should work offline; we don’t have stable internet.” (Surgeon, Public Hospital, P19)
74.6 % interested in AI training	High motivation despite barriers	“We need AI training; it is the future of surgery.” (Surgeon, Public Hospital, P15)
66.7 % believed AI could help triage	AI as lifesaving triage tool in mass casualties	“AI could help prioritize patients in mass casualty events.” (Surgeon, Public Hospital, P01)

Footnote:.

Quantitative findings are derived from survey responses (*n* = 185) and indicate the proportion of participants reporting each barrier or perception. Qualitative themes were identified through thematic analysis of semi-structured interviews and provide contextual explanation of the survey results. Representative quotes illustrate participants’ perspectives and highlight how infrastructure, training, financial, and conflict-related constraints influence AI adoption in Sudanese surgical practice.

## 4 Discussion

### 4.1 Global context and comparative insights

This study provides a real-world assessment of AI adoption in surgical care from an active conflict zone in Africa. Prior research in LMICs has largely focused on radiology or public health in stable environments [17]. In contrast, our findings reflect frontline surgical perspectives from war-affected hospitals across multiple sectors. While AI awareness was moderate, hands-on use and training were minimal—consistent with global trends but intensified by Sudan’s infrastructure collapse and displacement of surgical teams. These conditions require offline-capable, low-bandwidth AI tools. Our findings offer a foundation for reimagining AI as a tool for surgical resilience in conflict-affected and fragile settings worldwide.

### 4.2 Role of AI in cardiac and thoracic surgery

Recent systematic reviews delineate AI's established role in enhancing precision and decision-making in cardiac and thoracic surgery in stable settings, primarily through robotic assistance, advanced imaging analysis, and predictive analytics for complications [18,19]. Our findings from Sudan present a congruent vision of AI's perceived utility but within a radically different implementation context. In Sudan, surgeons recognize similar potential, but severe infrastructure gaps and limited training dominate. Unlike resource-rich settings where AI augments capacity, in conflict-affected environments it must first address systemic deficits. Therefore, priorities in triage and diagnostics reflect a distinct entry point, emphasizing the need for robust, offline-capable tools before deploying advanced surgical AI.

### 4.3 AI-Enhanced telemedicine and remote surgical support

In conflict-affected regions, the scarcity of specialized medical personnel poses a substantial challenge. AI-driven telemedicine platforms have been used to bridge this gap by facilitating remote consultations and surgical guidance. For example, during the Syrian conflict, organizations implemented tele-ICU programs where off-site specialists supported local medical staff through digital interfaces. Integrating AI into these platforms can further improve diagnostic accuracy and clinical recommendations, especially in triage and acute care scenarios [20]. In Sudan, where surgical teams are fragmented and infrastructure is damaged, surgeons supported AI for perioperative care. Many emphasized the urgent need for mobile-based telemedicine systems with embedded decision support—particularly to compensate for the absence of radiologists and pathologists in rural or besieged regions. These tools could sustain surgical care during active crises by enabling remote consultations and bridging workforce gaps [21].

### 4.4 AI-Assisted diagnostic imaging

Delays in radiology reporting and limited specialist access frequently hinder emergency surgery in conflict settings. AI-powered imaging and advanced image-processing techniques have demonstrated substantial accuracy in analysing computed tomography (CT) and magnetic resonance imaging (MRI), enabling early detection, classification, and prognostic assessment of complex pathologies such as liver cancer [22]. These advances highlight the broader potential of AI to compensate for specialist shortages by rapidly interpreting imaging data. In acute care scenarios, AI-driven tools can also identify critical trauma patterns—such as internal bleeding or fractures—thereby enhancing triage and surgical decision-making. In Ukraine, battlefield AI systems have supported faster trauma interpretation despite scarce radiology services [23].

For Sudan, AI-assisted CT analysis (e.g., detection of intra-abdominal hemorrhage) could similarly reduce reliance on unavailable radiology specialists and facilitate earlier surgical intervention, acting as a diagnostic bridge in war-affected hospitals where time and resources are severely constrained [24].

### 4.5 AI for surgical training and simulation

Armed conflict often disrupts traditional surgical training, creating a dangerous gap in workforce development. AI-powered simulation tools offer a scalable solution, enabling trainees to practice procedures virtually without needing onsite mentorship. The David Nott Foundation has used such models in Ukraine to strengthen local surgical capacity under war conditions [25].

In Sudan, where many residency programs are suspended or displaced, AI-based simulation could help preserve core competencies. Integrating these tools into emergency training curricula may sustain surgical skills and ensure continuity of care even in prolonged crises [26].

### 4.6 Systemic barriers and ethical considerations

Adoption of AI in Sudanese surgery is hindered by structural barriers—chiefly limited infrastructure (87.6 %), lack of training (79.1 %), and financial constraints (72.4 %). Nearly half of participants (48.9 %) also raised ethical concerns, including reliability, bias, and accountability for machine-assisted decisions [24].

These issues are intensified by conflict-related challenges, such as hospital attacks and the collapse of digital infrastructure. Broader concerns about AI misuse—such as disinformation campaigns observed in Syria, Myanmar, and Ethiopia—underscore the need for safeguards in fragile contexts [[27], [28], [29]]. For AI to support surgical care equitably, implementation must be transparent, culturally informed, and co-designed with frontline providers. Otherwise, digital interventions risk exacerbating, rather than alleviating, systemic health disparities.

### 4.7 Training needs and local readiness

Our findings reveal a critical paradox: Sudanese surgeons are eager to adopt AI, yet exposure remains minimal. In this study, 74.6 % expressed interest in AI training and 68.2 % supported its inclusion in surgical curricula, but only 9.4 % had hands-on experience and 10.3 % were familiar with advanced AI concepts. Junior residents showed the lowest familiarity, highlighting a training-level-dependent gap.

This underscores the need to embed AI literacy early in surgical education and to develop scalable, offline-compatible simulation tools suited to fragile health systems, ensuring continuity despite infrastructural or geopolitical disruptions. The observed ‘Seniority Gap’—where senior residents acquire AI skills through necessity while 1st- and 2nd-year trainees lack foundational knowledge—calls for formal integration of AI training into residency programs, transitioning from ad-hoc experiential learning to structured competency development.

### 4.8 Global collaboration for humanitarian AI deployment

AI has the potential to support triage, risk prediction, and decision-making in conflict-affected hospitals. Yet Sudan’s crisis—marked by hospital attacks, staff displacement, and infrastructure collapse—requires tools that are offline-capable, locally adapted, and language-accessible.

Global collaboration is essential. Humanitarian organizations can pilot AI in mobile clinics, while satellite providers may help address connectivity gaps. Donor agencies must prioritize open-source, ethically designed technologies tailored to fragile settings. Without coordinated investment, AI may widen, rather than bridge, global surgical disparities.

### 4.9 Comparison with other low-resource and conflict settings

In conflict settings like Ukraine and Syria, AI has supported trauma triage and remote diagnostics through battlefield algorithms and telemedicine platforms [30]. However, Sudan’s collapse presents unique challenges. Unlike Ukraine—where hospitals retain digital infrastructure—many Sudanese facilities have been destroyed or disconnected entirely, with limited access to global AI networks due to political isolation.

Sudan’s situation, however, is more extreme: many hospitals are non-functional, digital access is unreliable, and global integration is hindered by political isolation.

These challenges underscore the need for AI tools specifically designed for offline, low-bandwidth use. Sudan illustrates that conventional AI models cannot be simply adapted—they must be rebuilt for fragility.

This study offers one of the first comparative perspectives on AI in war-affected surgical systems. While Sudanese surgeons reported slightly lower AI awareness (68.7 %) than regional averages, their enthusiasm for training (74.6 %) affirms readiness despite collapse [31,32].

### 4.10 Proposed implementation models

Drawing on our findings, we propose a context-adapted AI framework to meet the demands of surgical care in war-affected, low-resource settings like Sudan. The model includes three core domains:

• Emergency Triage and Prioritization
• Complication Risk Prediction
• Telemedicine-Enabled Surgical Support

These components are not standalone; they must be integrated into a scalable, offline-compatible ecosystem responsive to both resource constraints and local clinical realities.

To operationalize AI use in extreme conditions, we propose a practical, adaptable model as shown in (Supplementary file 6).

### 4.11 Limitations and future research directions

This study has several limitations. First, data collection through online platforms may have introduced selection bias, as surgeons in fully besieged or severely resource-limited areas were less likely to be reachable. Participants who completed the survey were more likely to have at least intermittent internet access and institutional support, which may be associated with greater exposure to digital tools; therefore, levels of AI awareness and readiness may be slightly overestimated. Second, self-reported measures of AI familiarity and readiness may be subject to measurement bias, potentially over- or underestimating actual experience and competence with AI tools. Third, the study period (October 2024–June 2025) coincided with an evolving conflict, and timing effects may have influenced responses due to changes in security, resource availability, and clinical workload over time. Fourth, senior consultants were underrepresented, limiting the inclusion of institutional and policy-level perspectives. Finally, generalizability beyond Sudan may be limited, as political isolation, variable infrastructure, and the dynamic nature of conflict may affect the feasibility and acceptance of AI in other low-resource or war-affected settings. The rapidly changing conflict context may also affect applicability over time, as access to technology, internet connectivity, and healthcare delivery systems may shift rapidly.

Future research should include prospective pilot studies deploying offline and context-adapted AI tools in conflict-affected hospitals to assess clinical impact on surgical decision-making. Engaging both clinicians and patients in co-design will ensure tools are contextually appropriate, acceptable, and ethically sound. There is also a need for validated ethical frameworks to guide equitable, scalable AI implementation in fragile health systems. Additionally, patient perspectives should be actively included to evaluate trust, informed consent, and ethical implications of AI-assisted surgical care, particularly in regions with low health literacy and ongoing conflict.

## 5 Conclusion

This study provides the first evidence from an African conflict zone assessing the feasibility of artificial intelligence in surgical care. Despite Sudan’s profound healthcare collapse, surgeons demonstrated strong interest in AI-supported triage, diagnostics, and decision-making, even amid limited infrastructure and training. The results highlight that AI is not a luxury in such settings, but a potential lifeline—especially when tailored to offline, low-bandwidth environments. To advance surgical resilience in war-affected regions, investment must shift toward ethically guided, context-specific AI tools that align with the realities of fragile health systems. These findings should inform national surgical plans, training curricula, and global health strategies seeking to future-proof surgical care in humanitarian crises.

## Funding

This research did not receive any specific grant from funding agencies in the public, commercial, or not-for-profit sectors.

## Informed consent and ethical approval

This study was approved by the Research Ethics Committee of the Gezira State Ministry of Health. All participants provided written informed consent before participation. No patient-identifiable information, images, or videos are included in this manuscript. Where applicable, all efforts were made to ensure anonymity and confidentiality.

## Use of generative AI in the writing process

During the preparation of this work, the authors used generative AI tools (ChatGPT 3, OpenAI) to enhance clarity and language only. After using this tool, the authors reviewed and edited the content as needed and take full responsibility for the content of the publication.

## Sex and gender-based analysis

This study involved adult surgical professionals and did not include clinical or biological outcomes from human or animal subjects. Therefore, sex- and gender-based analyses were not applicable. This is acknowledged as a limitation in generalizability, and future studies incorporating patient-level perspectives may address this gap.

## Sources of financial support

None

## CRediT authorship contribution statement

**Alsadig Suliman:** Writing – review & editing, Writing – original draft, Visualization, Validation, Supervision, Software, Resources, Project administration, Methodology, Investigation, Funding acquisition, Formal analysis, Data curation, Conceptualization. **Lina SeedAhmed:** Validation, Formal analysis, Data curation. **Sara Hussein:** Writing – original draft, Software, Resources. **Siddig Ali:** Investigation, Data curation. **Sabah Ahmed:** Visualization, Funding acquisition, Formal analysis. **Alkhansa Alkhider:** Resources, Investigation.

## Declaration of competing interest

The authors declare that they have no known competing financial interests or personal relationships that could have appeared to influence the work reported in this paper.

## Appendix Supplementary materials

Image, application 1

Image, application 2

Image, application 3

Image, application 4

Image, application 5

Image, application 6
