# Supplementary material for: Artificial intelligence for surgical care in war-torn sudan: Feasibility, barriers, and ethical perspectives from a conflict zone
Source: Surg Pract Sci. 2026 Feb 15;25:100333. doi: 10.1016/j.sipas.2026.100333 (PMC12937154; doi:10.1016/j.sipas.2026.100333)
Supplement: Supplementary file 1 [file mmc1.docx]

| **Domain** | **Source** | **N** | **Type** | **Example Questions** |
| --- | --- | --- | --- | --- |
| 1.Demographic Data | Adapted from ARIES Survey | 5 | Multiple-choice | Age, gender, surgical experience, practice setting, conflict-zone experience |
| 2. AI Awareness & Familiarity | ARIES Survey + Adaptation | 6 | Multiple-choice + Open-ended | Are you familiar with AI terms (General AI, Narrow AI, Machine Learning, Deep Learning, Supervised/Unsupervised Learning, Computer Vision, NLP)? Have you used AI-based tools in practice? Define AI in your own words. |
| 3. Perceived Barriers | ARIES Survey | 5 | Multiple-choice | Limited infrastructure, lack of training, financial constraints, resistance from senior staff, lack of guidelines |
| 4. Ethical & Regulatory Concerns | Saudi AI Awareness Questionnaire | 10 | 5-point Likert scale | AI bias concerns; trust in AI recommendations; patient data privacy; fear of over-reliance; accountability in surgical errors |
| 5. Professional Impact Perceptions | Saudi Questionnaire + Adaptation | 4 | 5-point Likert scale | AI replacing clinical judgment; impact on surgical training; anxiety about technology adoption; professional identity concerns |
| 6. AI in War-Affected Settings | Newly developed | 4 | Multiple-choice + Open-ended | Can AI improve triage and emergency surgical care in conflict zones? What barriers are unique to war-affected hospitals? |
| 7. Usefulness of AI in Emergency Surgery | Combined (ARIES + Saudi) | 7 | Multiple-choice | Perioperative decision-making, intraoperative decision-making, improved surgical vision, surgical practice, training and education, surgical robot automation, high-tech surgical devices |
| 8. Readiness & Willingness to Use AI | (ARIES + Saudi + UTAUT adaptation) | 2 | 5-point Likert scale | Willingness to attend AI training; support integrating AI in surgical education |
| 9. Suggestions, Comments | ARIES (Open-ended) | 2 | Open-ended | What suggestions or concerns do you have regarding AI adoption in Sudanese surgery? |

**Footnote:**

*This table summarizes the survey domains, the source of each domain, the number and type of items, and representative example questions. The survey included both multiple-choice and open-ended items, with some questions adapted from validated instruments (ARIES Survey, Saudi AI Awareness Questionnaire) and others developed to address AI application in war-affected surgical settings. This table presents the questionnaire structure only; responses are reported separately in the results section*

**Supplementary Material 1:** Questionnaire Structure. Domains, Sources, and Example Questions of the Survey Instrument on Artificial Intelligence in Surgical Care
