# Supplementary material for: Artificial intelligence for surgical care in war-torn sudan: Feasibility, barriers, and ethical perspectives from a conflict zone
Source: Surg Pract Sci. 2026 Feb 15;25:100333. doi: 10.1016/j.sipas.2026.100333 (PMC12937154; doi:10.1016/j.sipas.2026.100333)
Supplement: Supplementary file 2 [file mmc2.docx]

**Supplementary table 2**: Sample calculation

To calculate the total population (N) from a known sample size (n) using Cochran's formula with a finite population correction, we use the following steps:

**1. Total Population Calculation**

Given:

- Sample Size (n): 195
- Confidence Level: 95% (Z = 1.96)
- Margin of Error (e): 5% (0.05)
- Variability (p): 0.5 (maximum variability assumed)

First, we find the ideal sample size for an infinite population (no):


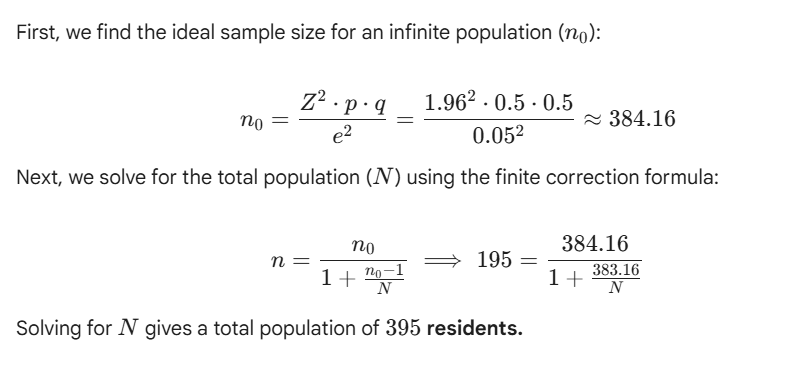


Next, we solve for the total population (N) using the finite correction formula:


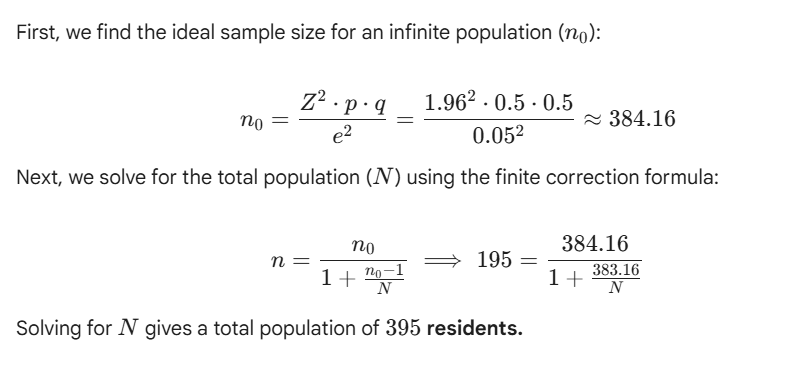


Solving for N gives a total population of 395 residents.

**2. Population per Patch (Proportional Allocation)**

Assuming the sample sizes per patch were chosen proportionally to the actual population sizes, here is the breakdown of the population for each patch:

| **Patch ID** | Sample Size (n) | Estimated Population (N₂) |
| --- | --- | --- |
| **Patch 43** | 9 | 18 |
| **Patch 44** | 28 | 57 |
| **Patch 45** | 25 | 51 |
| **Patch 46** | 26 | 53 |
| **Patch 47** | 19 | 38 |
| **Patch 48** | 21 | 43 |
| **Patch 49** | 23 | 47 |
| **Patch 50** | 22 | 45 |
| **Patch 51** | 22 | 45 |
| **Total** | **195** | 397* |
