# Supplementary material for: Artificial intelligence for surgical care in war-torn sudan: Feasibility, barriers, and ethical perspectives from a conflict zone
Source: Surg Pract Sci. 2026 Feb 15;25:100333. doi: 10.1016/j.sipas.2026.100333 (PMC12937154; doi:10.1016/j.sipas.2026.100333)
Supplement: Supplementary file 3 [file mmc3.docx]

# Supplementary File 3: Interview Guide Interviewer: (Same for all Process) Estimated Duration: 25–35 minutes

# Mode: Phone/Zoom/In-person

## A. Opening Script

Thank you for agreeing to participate. The purpose of this interview is to understand perceptions and experiences regarding artificial intelligence (AI) in surgical care in Sudan, particularly in conflict-affected settings. Your responses will be anonymized, and no identifiable information will be reported.

**Do you consent to participate and be recorded?** Only If yes, proceed.

## B. Background and Context

**1.** Please describe your current role and the type of hospital or facility where you work.

**2.** Have you worked in conflict-affected or partially accessible regions? If yes, please describe the main challenges you face.

## C. AI Awareness and Experience

**3.** What is your understanding of AI in medicine or surgery?

**4.** Have you ever used any AI tool in clinical practice? If yes, which tools and in what context?

## D. Perceived Benefits

**5.** In your opinion, what are the potential benefits of AI in surgical care in Sudan?

**6.** Which areas would benefit most (e.g., triage, diagnostics, preoperative planning, intraoperative support, postoperative monitoring, training)?

## E. Barriers and Challenges

**7.** What are the main barriers to AI adoption in your setting? (Probe: infrastructure, training, costs, institutional resistance, ethics)

**8.** How does the conflict environment affect AI feasibility (power outages, internet connectivity, security, equipment damage, displacement)

## F. Ethical and Governance Concerns

**9.** What ethical concerns do you have regarding AI in surgery? (Probe: accountability, bias, consent, data privacy)

**10.** Who should be responsible for AI-related errors or adverse outcomes?

**11.** How should patient consent and data protection be handled when using AI tools in your setting?

## G. Future Directions and Recommendations

**12.** What kind of AI tools would be most useful in your context (offline tools, mobile apps, imaging support, triage systems)?

**13.** What training, infrastructure, or policy support is needed to enable AI adoption?

**14.** Do you have any final thoughts or recommendations regarding AI integration in surgical care in Sudan?

*End of Interview Guide.*
