# Supplementary material for: Artificial intelligence for surgical care in war-torn sudan: Feasibility, barriers, and ethical perspectives from a conflict zone
Source: Surg Pract Sci. 2026 Feb 15;25:100333. doi: 10.1016/j.sipas.2026.100333 (PMC12937154; doi:10.1016/j.sipas.2026.100333)
Supplement: Supplementary file 4 [file mmc4.docx]

**Supplementary Table 4**: Associations Between Participant Characteristics and AI Readiness

| Predictor Variable | Categories | Total N (%) | AI Ready n (%) | Test Statistic | p-value | VIF* |
| --- | --- | --- | --- | --- | --- | --- |
| Age | 24–29 | 45 (24.3%) | 25 (55.6%) | H=171.61 | <0.001 | 8.42 |
|  | 30–34 | 72 (38.9%) | 50 (69.4%) |  |  |  |
|  | ≥35 | 68 (36.8%) | 55 (80.9%) |  |  |  |
| Sex | Male | 132 (71.4%) | 85 (64.4%) | χ2=3.20 | 0.074 | 1.15 |
|  | Female | 53 (28.6%) | 40 (75.5%) |  |  |  |
| Training Level | 1st–2nd Year | 56 (30.3%) | 25 (44.6%) | H=171.61 | <0.001 | 7.91 |
|  | 3rd–4th Year | 63 (34.1%) | 42 (66.7%) |  |  |  |
|  | 5th Year | 66 (35.6%) | 58 (87.9%) |  |  |  |
| Hospital Type | Public | 116 (62.7%) | 70 (60.3%) | χ2=5.82 | 0.121 | 1.44 |
|  | Military/NGO | 29 (15.7%) | 23 (79.3%) |  |  |  |
|  | Private | 7 (3.8%) | 5 (71.4%) |  |  |  |
|  | Other/Displaced | 33 (17.8%) | 22 (66.7%) |  |  |  |
| Prior AI Training | Yes | 41 (22.2%) | 38 (92.7%) | χ2=38.51 | <0.001 | 2.1 |
|  | No | 144 (77.8%) | 87 (60.4%) |  |  |  |
| Advanced Literacy | Yes | 19 (10.3%) | 18 (94.7%) | χ2=21.84 | <0.001 | 2.33 |
|  | No | 166 (89.7%) | 107 (64.5%) |  |  |  |

**Notes:**

- AI readiness defined as participant confidence and willingness to adopt AI in clinical practice.
- χ² (chi-square) used for categorical variables, Kruskal-Wallis (H) for ranked/continuous predictors.
- Multivariable regression was explored but not retained due to collinearity and small sample size for some predictors.
