# Supplementary material for: Artificial intelligence for surgical care in war-torn sudan: Feasibility, barriers, and ethical perspectives from a conflict zone
Source: Surg Pract Sci. 2026 Feb 15;25:100333. doi: 10.1016/j.sipas.2026.100333 (PMC12937154; doi:10.1016/j.sipas.2026.100333)
Supplement: Supplementary file 5 [file mmc5.docx]

# Supplementary File 5: Codebook

*This codebook is intended to guide thematic analysis of semi-structured interviews regarding artificial intelligence (AI) in surgical care in Sudan. It defines codes, descriptions, inclusion/exclusion criteria, and example quotes.*

| **Code** | **Code Name** | **Definition** | **Inclusion / Exclusion** | **Example Quote** |
| --- | --- | --- | --- | --- |
| A1 | AI Knowledge and Awareness | Understanding of AI, definitions, and familiarity with AI concepts in healthcare. | Includes: basic AI concepts, examples of AI tools, awareness of AI in medicine. Excludes: general digital technology (non-AI). | “I know AI as a system that can analyze images and suggest diagnoses.” |
| A2 | Current Use of AI/Technology | Actual or prior use of AI or digital tools in clinical practice. | Includes: use of AI in imaging, decision support, telemedicine, mobile apps. Excludes: hypothetical use without experience. | “We use WhatsApp for consultations, but not AI yet.” |
| B1 | Perceived Benefits | Perceived advantages of AI for surgical care. | Includes: improved diagnostics, triage, training, efficiency, decision support. Excludes: benefits unrelated to AI (e.g., general staff increases). | “AI could help with preoperative planning when specialists are not available.” |
| B2 | Priority Areas for AI | Clinical or operational areas where AI is most needed. | Includes: triage, imaging, monitoring, training, supply chain. Excludes: non-surgical domains unless directly relevant. | “Triage in the emergency department would benefit most.” |
| C1 | Infrastructure Barriers | Physical and technical limitations to AI implementation. | Includes: electricity, internet, hardware, maintenance. Excludes: purely ethical or policy barriers. | “Power cuts and poor internet make it impossible to rely on AI.” |
| C2 | Training and Human Capacity | Staff skills, education, and willingness to use AI. | Includes: need for training, resistance to change, skill gaps. Excludes: infrastructure issues. | “Surgeons need training to interpret AI outputs.” |
| C3 | Cost and Resource Constraints | Financial and resource-related barriers. | Includes: device costs, licensing, funding, competing priorities. Excludes: non-financial barriers. | “We cannot afford AI systems with limited budgets.” |
| C4 | Conflict-Specific Constraints | Challenges unique to conflict settings. | Includes: security, displacement, damaged facilities, supply disruptions. Excludes: general low-resource settings without conflict. | “When the hospital is bombed, equipment gets destroyed.” |
| D1 | Ethical Concerns | Ethical issues raised by AI use. | Includes: bias, consent, transparency, accountability. Excludes: operational issues. | “AI may be biased against our population because it was trained elsewhere.” |
| D2 | Data Privacy and Security | Concerns about patient data protection. | Includes: data storage, sharing, confidentiality, cyber risks. Excludes: general privacy unrelated to AI. | “We worry about patient data being leaked.” |
| D3 | Accountability and Liability | Who is responsible for AI errors. | Includes: clinician responsibility, manufacturer liability, institutional governance. Excludes: general legal issues not tied to AI. | “If AI makes a wrong decision, who is blamed?” |
| E1 | Governance and Policy Needs | Need for regulation, guidelines, and institutional frameworks. | Includes: national policies, hospital protocols, ethics committees. Excludes: individual-level opinions without policy implications. | “We need a national guideline for AI use in hospitals.” |
| E2 | Implementation Strategies | Practical steps to introduce AI. | Includes: pilot projects, partnerships, capacity building, phased rollout. Excludes: abstract recommendations without action steps. | “Start with a small pilot in one hospital.” |
| E3 | Preferred AI Characteristics | Desired features for AI tools. | Includes: offline functionality, local language support, low bandwidth, user-friendly interface. Excludes: generic tech preferences not specific to AI. | “We need AI that works offline due to power outages.” |
| F1 | Perceived Risks and Unintended Consequences | Potential harms or negative outcomes. | Includes: overreliance, deskilling, misuse, inequity. Excludes: benefits. | “Doctors may rely on AI and lose their skills.” |
| F2 | Trust and Acceptance | Degree of trust in AI and willingness to use it. | Includes: skepticism, trust-building factors, cultural acceptance. Excludes: technical barriers only. | “I do not trust AI unless I see evidence.” |
| G1 | Patient and Community Perspectives | Views on AI from patients/community (if mentioned). | Includes: patient fears, expectations, consent attitudes. Excludes: clinician-only perspectives. | “Patients may fear their data is used without permission.” |
| H1 | Research and Evidence Needs | Need for evidence, evaluation, and research. | Includes: need for local studies, validation, monitoring outcomes. Excludes: general statements without evidence focus. | “We need trials to show AI works here.” |
