# Supplementary material for: Artificial intelligence for surgical care in war-torn sudan: Feasibility, barriers, and ethical perspectives from a conflict zone
Source: Surg Pract Sci. 2026 Feb 15;25:100333. doi: 10.1016/j.sipas.2026.100333 (PMC12937154; doi:10.1016/j.sipas.2026.100333)
Supplement: Supplementary file 6 [file mmc6.docx]

**Supplementary Material 6**. Strategies for Context-Adapted AI Implementation in Conflict-Affected Surgical Settings

| **Component** | **Strategy** |
| --- | --- |
| **Offline & Edge Computing** | Edge-based AI ensures functionality without constant internet; frameworks like Petri-net-supported systems have shown reliability in low-bandwidth Eds. |
| **Satellite & Intermittent Connectivity** | Leverage services like Starlink for remote updates, while core AI runs locally. |
| **Contextual Tools** | Mobile CT and telepathology tools tailored for trauma imaging and localized languages. |
| **Cultural Adaptation** | Interface and training in Sudanese Arabic to ensure usability and trust. |
| **Simulation-Based Surgical Training** | Low-fidelity surgical simulators with AI feedback can sustain training where mentorship is disrupted . |
| **Open-Access & Ethical Design** | Community-involved development based on global best-practices promotes sustainability and equity . |
